# Swinging rhythms

**DOI:** 10.1007/s12471-022-01694-8

**Published:** 2022-05-10

**Authors:** J. Dijkmans, J.-T. Wijmenga, R. Tukkie

**Affiliations:** 1 Department of Cardiology, Amsterdam University Medical Centre, location VUmc, Amsterdam, The Netherlands; 2 Department of Cardiology, Spaarne Hospital, Haarlem, The Netherlands

A 78-year-old woman receiving treatment for hypertension was admitted for diuretic therapy and rhythm monitoring after being diagnosed with congestive heart failure and atrial fibrillation. She was referred with progressive peripheral oedema and her chest radiograph showed signs of left-sided decompensation and pleural effusion. The transthoracic echocardiogram revealed severe dilated cardiomyopathy with a left ventricular ejection fraction of < 10% and severe tricuspid regurgitation.

The initial electrocardiogram (Fig. 1) showed atrial fibrillation with a ventricular rate of 168 bpm and normal QRS duration. After administration of digoxin with a total loading dose of 1 mg the ventricular rate decreased to 120 bpm. Eight hours later a broad complex tachycardia appeared on telemetric monitoring. The acquired electrocardiogram is presented in Fig. 2. Laboratory analyses revealed no evidence for electrolyte disturbances or myocardial injury. The measured digoxin plasma concentration was 1.3 µg/l (range 0.5–2.0).

**Fig. 1:**
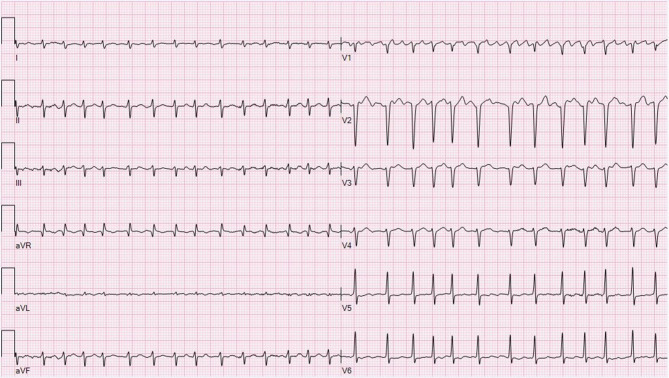
Electrocardiogram at presentation showing atrial fibrillation

**Fig. 2:**
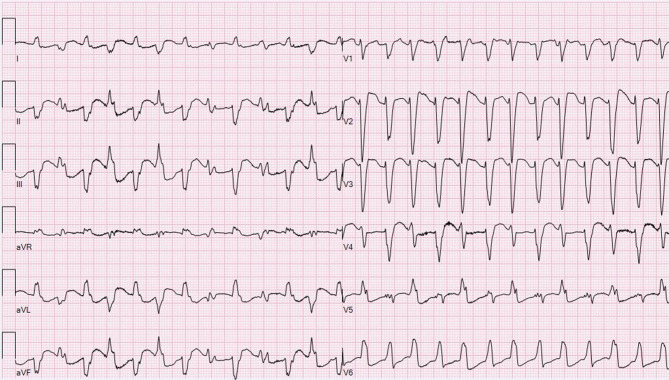
Electrocardiogram showing broad complex tachycardia

What is your diagnosis? What is the most likely mechanism?

## Answer

You will find the answer elsewhere in this issue.

